# The Successful Conservative Treatment of Early Gas Gangrene in Limbs by the Resection of Infected Muscles

**Published:** 1917-12

**Authors:** 


**The Successful Conservative Treatment of Early Gas Gangrene in Limbs by the Resection of Infected Muscles.** By Lieut.-Col. C. H. S. Frankau, Capt. Hamilton Drummond, and Capt. G. E. Nelligan, all R. A. M. C. Abs. from the *British Medical Journal*, June 2, 1917.

The authors have based their work upon the observations made by Wallace (see p. 107) especially to the effect that gas gangrene is chiefly a disease of the muscles, that it rarely invades all the muscles of a limb, except in a segment completely cut off from blood supply, that it progresses longitudinally, that there is little tendency for the infection to pass from one muscle to another. The authors believe that these points cannot be controverted.

They have made it a point first to explore the primary focus so as to arrest infection by a resection of the infected areas. Such resection may involve a part or the whole of single muscles or groups of muscles.

Resection should be limited, however, to limbs in which the main blood vessel is intact, and which would be worth more than an artificial limb when saved; otherwise amputation should be preferred. Resection of large groups of muscle is, to be chosen, nevertheless, if amputation involves too great a risk to life. The resection should extend until muscle is reached with *normal color*, *normal contractibility*, and *a good blood supply*. Even if there is still infection in such muscles, the opening of the wound and free drainage arrests further development.

After resection, the following treatment is applied. (1) The dressings are reduced to the minimum — that is, one or two layers of gauze are placed over the wound so as to allow free access of air, and, if possible, sunshine to the wound region; (2) constant or intermittent irrigation of the wound by some modification of the Carrel method — eusol, saline, or hydrogen peroxide being used as the irrigating fluid.

The authors detail 14 cases in which such treatment has given excellent results. They call attention especially to one case in which the removal of only half of a muscle was necessary, because the infection showed no disposition to progress transversely. They also cite several cases in which generous resection took the place of amputations that would surely have proved fatal.

